# *Vitreoscilla filiformis* Supernatant: A Novel Postbiotic Secretome for the Prevention and Treatment of Wound Infections

**DOI:** 10.3390/pharmaceutics18060686

**Published:** 2026-05-30

**Authors:** Miranda Piccioni, Giuseppe Curcio, Alessandro Graziani, Donatella Pietrella

**Affiliations:** 1 Biochemical Sciences and Health Unit, Department of Pharmaceutical Sciences, University of Perugia, Via del Giochetto, 06122 Perugia, Italy; 2 Microbiology and Clinical Microbiology Section, Department of Medicine and Surgery, University of Perugia, 06129 Perugia, Italy

**Keywords:** *Vitreoscilla filiformis* supernatant, anti-biofilm, regenerative properties, wound infection treatment

## Abstract

**Background**: Biofilms consist of complex microbial communities embedded in an extracellular matrix which confer resistance to the most used antimicrobial agents. Chronic wounds are often associated with burns, trauma, surgery, diabetes and peripheral vascular disease. They are characterized by a marked delay in wound healing favoring the development of microbial biofilms, which in turn further delay tissue regeneration. *Staphylococcus aureus*, *Staphylococcus epidermidis*, and methicillin-resistant staphylococci biofilms are found in chronic wounds, seriously hindering wound treatment. *Vitreoscilla filiformis*, a Gram-negative non-pathogenic filamentous bacterium, has been shown to improve atopic dermatitis by reducing *S. aureus* colonization and inducing antioxidant responses in the skin. **Objectives**: The aim of the present study was to evaluate the antimicrobial, anti-inflammatory, and regenerative activities of the *V. filiformis* supernatant (VFS). **Methods**: The effect of VFS on bacteria growth was assessed by microbial growth kinetics and biofilm formation and dispersal. Antioxidant potential was determined by DPPH-scavenging ability and reduction in intracellular reactive oxygen species (ROS). The regenerative properties were assessed by scratch assay. **Results**: *V. filiformis* VFS holds strong anti-biofilm activity against *S. aureus*, *S. epidermidis* and methicillin-resistant *S. aureus* (MRSA), acting during both biofilm formation and dispersion. The decrease in biofilm mass is accompanied by a significant increase in the planktonic form compared to the untreated cells. Moreover, VFS is characterized by an interesting antioxidant activity, as demonstrated by a cell-free DPPH assay and a neutrophil-based in vitro assay. In addition, VFS can stimulate tissue regeneration in human dermal fibroblasts and keratinocytes. **Conclusions**: The demonstration of anti-biofilm, antioxidant and regenerative properties of *V. filiformis* supernatant could be exploited for the treatment of biofilm-associated wound infections.

## 1. Introduction

Human skin, covering roughly 30 m^2^ on average, provides a vast habitat for a diverse community of microorganisms. Sampling shows that the number of these microorganisms can range from 10^3^ to 10^6^ per site [1]. Notably, its bacterial population is mainly derived from four key phyla, *Actinomycetota*, *Bacillota*, *Pseudomonadota* and *Bacteroidota*, with genera such as *Staphylococcus*, *Cutibacterium*, *Corynebacterium*, *Micrococcus*, *Streptococcus* and *Acinetobacter* being particularly prevalent [2].

Human skin is colonized by millions of bacteria who are primarily acquired during birth and continuously subjected to deep modifications during life. Under “steady-state” conditions, host factors and interactions between microorganisms define a relative stability of the microbial communities preventing colonization by pathogens [3]. These bacterial communities form the skin microbiota, a critical contributor to host health by enhancing immune defenses and promoting tissue regeneration [4]. However, when dysbiosis occurs, bacteria that normally reside in the skin as commensals may become pathogenic and colonization by pathogens might be favored.

Changes in the composition of skin microbiota have been demonstrated to occur in many cutaneous diseases. In atopic dermatitis (AD), the proportion of *Staphylococcus epidermidis* and *Staphylococcus aureus* significantly increases during disease flares [5]; the proliferation of staphylococci is partly attributed to their ability to bind to a compromised skin barrier, such as fibronectin in the upper epidermis, which enhances *S. aureus* colonization in AD patients’ skin [6]. Furthermore it has been demonstrated that severity of AD correlates with the presence of different *Staphylococcus* species [7]. Saheb Kashaf S. et al. characterized the predominance of *Staphylococci* during AD at three defined time points, flare (skin disease exacerbation without recent therapies), post-flare and baseline, in a cohort of 83 AD subjects and 15 healthy controls. The results showed that among the four most prominent genera present on the skin at affected disease sites, only *Staphylococcus* showed a significant increase. A more detailed metagenomic analysis conducted from the skin swab samples describes how, at the infection site, most subjects (66%) are predominantly colonized by *S. aureus* or the closely related *Staphylococcus argenteus*, while 29% are predominantly colonized by *S. epidermidis* [8]. Moreover, it has been shown that the severity of AD correlates with the presence of *S. aureus*—biofilm producers—and that biofilm-growing cultures from AD isolates display a greater tolerance to antibiotics than planktonic-growing cultures, which could account for the bacterial re-colonization a few weeks after antibiotic treatment [9].

Another relevant skin disease is psoriasis, in which the skin microbiota affects the course of the disease. It is observed that the skin microbiota of individuals affected by psoriasis is substantially different from that of healthy subjects [10], with a marked reduction in its variability among those suffering from the condition compared to healthy individuals [11,12]. Both lesional and non-lesional plaques are enriched for *S. aureus* compared to healthy skin [13].

Polymicrobial colonization is a hallmark of both acute and chronic wounds, which may arise from burns, trauma, surgery, diabetes and peripheral vascular disease [14]. The analysis of swabs obtained from chronic wound infections in 163 patients showed that in 156 of them the culture test was positive. *Pseudomonas aeruginosa* was the dominant species (75 cases), with co-detections of *Klebsiella pneumoniae* (21 cases), *Staphylococcus aureus* (14 cases) and *Proteus mirabilis* (13 cases) [15]. Overall, chronic topical wounds commonly harbor concurrent infections by *S. aureus* and *P. aeruginosa* [16]; co-infection by these two bacterial species has been shown to impede wound healing, often extending closure times significantly compared to infections with only one species [17].

It has been demonstrated that *S. aureus* infection compromises wound healing by significantly prolonging the re-epithelialization phase.

Chronic wounds are often associated with the development of microbial biofilms, which contribute to the delay in wound healing typical of chronic wounds [18,19]. Evidence indicates that the prevalence of biofilm in wounds varies significantly depending on their condition: biofilm is present in 60% of chronic wounds, while it drops to 6% in acute wounds [20]. A pooled analysis of eight prospective cohort studies (185 chronic wounds) and one case report found that 78.2% of unhealed chronic wounds in humans contained biofilms [21].

Chronic wound biofilms form resilient mixed-species communities that hinder both epithelial and granulation tissue development and sustain a persistent low-level inflammatory state, all of which delay healing. Once established, this hidden microbial ecosystem can progressively undermine normal repair processes [22]. Bacterial biofilm can act by modulating the host’s immune response. Biofilms release planktonic bacteria, LPS, quorum-sensing signals, exotoxins and DNA, triggering neutrophil recruitment [23,24]. Recruited neutrophils are unable to carry out their function because the ROS produced cannot penetrate the Extracellular Polymeric Substances (EPS); at the same time, the presence of a biofilm hinders the normal clearance of neutrophils [25,26]. This leads to abnormal release of high levels of proteases that interfere with and delay the healing process [27].

Biofilms are microbial sessile communities in which microorganisms live attached to abiotic or biotic surfaces and communicate with each other via quorum sensing mechanisms. The extracellular matrix embedding cells is composed of proteins, lipids, nucleic acids and polysaccharides and confers resistance to antimicrobial agents and immune responses, rendering the microbial biofilm particularly difficult to treat compared to the planktonic counterpart [18]. The commensal *S. epidermidis* is one of the most commonly cultured bacteria in clinical microbiology laboratories among coagulase-negative staphylococci (CoNS), probably due to the presence of infection-associated genetic elements correlating with biofilm formation and methicillin resistance [28]; moreover, because *S. epidermidis* is ubiquitous on human skin, it often contaminates and thereby infects medical devices [29]. *S. aureus* wound infection represents the main cause of difficult-to-treat complications in Foot Diabetic Ulcers [30,31], particularly due to its activity in delaying wound healing [32]. The prevalence of methicillin-resistant *S. aureus* (MRSA) in foot ulcers is 15–30%, [33]. A recent study characterizing wound infections in 266 subjects found that 34.58% (92/266) were colonized by *S. aureus*; of those 28.3% (26/92) were MRSA [34].

The development of alternative biofilm eradication strategies is of pivotal importance for the treatment of skin infections.

*Vitreoscilla filiformis* is a non-pathogenic Gram-negative bacterium belonging to the order of *Beggiatoales*. This bacterium has been identified as “beneficial” for its positive effects on skin health. In clinical studies, *V. filiformis* biomass lysate could improve clinical signs in AD and reduce *S. aureus* colonization of the skin [35,36]. The protective effects on skin health could be due to the proven activation of skin major endogenous inducible free radical scavenger MnSOD (manganese superoxide dismutase), the stimulation of antimicrobial β-defensin production [36,37], and the induction of tolerogenic dendritic cells and Treg (regulatory T) cells able to suppress cutaneous inflammation [38]. Moreover, topical *V. filiformis* extract (VFE) exhibited biological activities comparable to those of probiotics, particularly in the immunomodulation of regulatory cells, protection against infection, and helping skin barrier function for better recovery and resistance [39]. The extract of *V. filiformis* cultured in Vichy volcanic mineralizing water (VVMW) showed an accelerated skin renewal and antioxidant activity [40]. VFE has been tested in clinical studies showing a significant improvement of AD due to reductions in *S. aureus* and to a direct immunomodulatory effect on skin [41].

The development of antibiotic resistance in cutaneous infections restricts the possibilities of treatment favoring the incidence of serious health complications. Building upon existing in vitro and in vivo studies on *V. filiformis* that have highlighted its potential use in therapeutic applications, particularly for skin-related conditions due to its immunomodulatory and anti-inflammatory properties, the aim of this study was to evaluate the beneficial biological activities of the *V. filiformis* (VF)-conditioned medium (supernatant, VFS), including its antioxidant potential, on biofilm formation by bacteria commonly associated with wound infections and cutaneous diseases, such as *P. aeruginosa*, *S. aureus*, *S. epidermidis*, and the clinical methicillin-resistant strain MRSA. In addition, we assessed the regenerative properties of the VFS on human dermal fibroblasts and human keratinocytes and explored its capacity to mitigate oxidative stress through antioxidant mechanisms.

## 2. Materials and Methods

### 2.1. Microbial Strains and Growth Conditions

Bacterial species involved in wound infections were selected. *Staphylococcus aureus* (ATCC 29213, American Type Culture Collection, Manassas, VA, USA), *Staphylococcus epidermidis* (ATCC 35984), *P. aeruginosa* (PAO-1, ATCC15692) and the clinical isolate methicillin-resistant *S. aureus* MRSA (collected during the routine clinical laboratory activity at the University Hospital of Perugia, Perugia, Italy) were initially streaked from −80 °C glycerol stock and maintained in tryptic soy agar (TSA, Sigma Aldrich-Merck Life Science, Milano, Italy). The bacterial cultures were grown to carry out the subsequent experiments of the study. The day before the test, one colony was inoculated in tryptic soy broth (TSB, Sigma Aldrich) and incubated for 24 h at 37 °C. Microbial cells were harvested by centrifugation, washed in Phosphate-Buffered Saline (PBS), counted by spectrophotometric analysis at 600 nm and resuspended to 10^6^ CFU/mL in the appropriate culture medium.

### 2.2. Vitreoscilla filiformis and Culture Supernatant VFS Preparation

*V. filiformis* was chosen based on its documented probiotic properties. The preparation of the bacterial culture and subsequent collection of the supernatant represent essential preliminary steps for evaluating the biological potential of VFS in downstream experimental assays. *V. filiformis* (ATCC^®^ 15551™) was purchased by LGC Standards (Milan, Italy); the deposited name is *Vitreoscilla filiformis* Strohl et al. (1986) [42]. *V. filiformis* was grown in Beggiatoa (ATCC^®^ medium: 138 Beggiatoa medium) at 37 °C; 0.1 mL was transferred to new media every 7 days. The day prior to the experiment, 7 mL TSB was inoculated with 100 µL of an overnight culture (dilution 1:70) and incubated for 18–24 h at 37 °C. The culture (1–1.5 × 10^8^ CFU/mL) was centrifuged at 3000 rpm for 10 min; the culture medium was filtered through a 0.45 µm filter (GVS Filter Technology, Bologna, Italy)) and used for downstream experiments.

### 2.3. Microbial Growth Kinetics

Growth kinetics assays were employed to demonstrate the viability of the initial (untreated) bacterial cultures and to assess the antimicrobial effect of VFS at varying concentrations and time points. In total, 200 µL of staphylococci and *P. aeruginosa* suspensions (10^5^ CFU/mL) was incubated in TSB at 37 °C, in the presence of different percentages of *V. filiformis* supernatant (VFS—1%, 5%, 25%, 75%, 90%, *v*/*v*) or gentamicin (2.5 µg/mL) as control, in Infinite 200 M pro microplate reader equipped with a monochromator (Tecan Italia S.r.l., Cinisello Balsamo, Italy). Absorbance at 600 nm was recorded every 60 min for a total of 24 h of incubation [43]. Each experiment was performed at least twice, and each sample was analyzed in triplicate.

### 2.4. Effect of V. filiformis Supernatant (VFS) on S. aureus, S. epidermidis and MRSA Biofilm Formation and Dispersal

The in vitro static biofilm assay was performed as previously described with some modifications [44]. Briefly, *S. aureus*, *S. epidermidis* and MRSA cultures were diluted overnight at 1:100 in TSB supplemented with 2% sucrose; 100 µL standardized bacterial cultures (1 × 10^6^ CFU/mL) were incubated in a flat-bottom 96-well plate at 37 °C for 24 h under static conditions in the presence or absence of different percentages (5–25–75% *v*/*v*) of VFS or gentamicin as a control [44]. To assess the capacity of VFS to disperse established biofilms, bacteria were cultured in TSB 2% sucrose for 24 h at 37 °C as previously described. Biofilms were treated with different percentages (1–5–25–75–90–100% *v*/*v*) of VFS for 4 h or 24 h at 37 °C. After incubation, the planktonic cultures were transferred to a new plate, and absorbance was read at 600 nm. For analyzing the biofilm mass, wells were washed twice with 200 µL of distilled water. Then, 50 µL of 0.4% Crystal Violet was added to each well for a minimum of 20 min. Wells were then washed twice with distilled water, and the Crystal Violet was resuspended by adding 100 µL of 90% ethanol and incubated at room temperature for 15 min. Absorbance was read at 570 nm. Bioassays were performed in triplicate in at least three separate experiments. The effect of VFS was assessed by comparing the percentage change in biofilm biomass with the proportion of planktonic bacteria, to distinguish its impact on the sessile bacteria versus free-living microbial populations.

### 2.5. DPPH Radical Scavenging Activity

The antioxidant activity of VFS was evaluated by using the 2,2-diphenyl-1-picrylhydrazyl (DPPH) free radical scavenging assay as described by Dutra et al. [45] with some modifications. DPPH is a stable free radical at room temperature, which can be reduced by the transfer of a hydrogen atom from an antioxidant agent, causing a change in color of DPPH from deep violet to yellow evaluable by spectrophotometry analysis. Because TSB showed some antioxidant activity, *V. filiformis* was cultured in 7 mL of Roswell Park Memorial Institute 1640 (RPMI 1640, Gibco-Thermo Fisher Scientific, Milan, Italy)). The day after, the culture was centrifuged at 3000 rpm for 10 min and the supernatant was filtered. Different percentages of *V. filiformis* supernatant were prepared (1–25–75% *v*/*v*) in RPMI 1640. The reaction was carried out on a flat-bottom 96-well plate in a final volume of 200 µL. The reaction mixture consisted of sample and DPPH in methanol (25 µg/mL). RPMI 1640 has been used as negative sample control. The control solution was prepared by mixing methanol and DPPH. A mixture of methanol and sample was used as blank. The absorbance was read at 517 nm in Infinite 200 M pro spectrophotometer plate reader (Tecan). Ascorbic acid (100 µg/mL) was used as positive control. Experiments were performed in triplicate.

The percentage of antioxidant activity (AA%) was calculated according to the following formula: AA%= 100 − [[(Abs sample − Abs blank) × 100]/Abs control].

### 2.6. Polymorphonuclear Cell (PMN) Isolation

Human heparinized venous blood was obtained from buffy coat provided by the Blood Bank of Ospedale della Misericordia of Perugia. Donors signed a consent form (MOSIT 06) approved by Ethics Committee CEAS (Comitato Etico Aziende Sanitarie) (Rev. 3 October 2014) in which they authorize the use of their sample for research studies. The blood was diluted with RPMI 1640 (Gibco) and subjected to density gradient centrifugation over Ficoll-Hypaque Plus (Pharmacia Biotech, Uppsala, Sweden) [46]. PMNs were recovered and washed twice. The pellet was treated with hypotonic saline solution to lyse contaminant erythrocytes and PMNs were suspended in RPMI 1640, washed twice, counted and diluted to the appropriate concentration. PMNs were chosen for their key role in inflammation and high ROS output, making them ideal for assessing oxidative stress modulation.

### 2.7. Evaluation of ROS Production by Luminol Assay

Assessing VFS-induced ROS reduction is key to evaluating its regenerative potential, since high ROS levels hinder wound healing. A total of 100 µL of cellular suspension (1.25 × 10^6^ cells/mL) was stimulated with different percentages of VFS (1–5–25% *v*/*v*) grown in RPMI 1640 in the presence of 50 µL of Luminol (final concentration 0.28 mM) and the mixture was incubated for 3 min at 37 °C as previously described [47]. The cells were then stimulated with 50 µL of PMA (phorbol-12-myristate-13-acetate), final concentration 100 ng/mL, and chemiluminescence was monitored for 20 min in a luminometer reader (TECAN). The light output was recorded as RLU (relative luminescence units). Each experiment was performed in triplicate.

### 2.8. Cytotoxicity Assay

The cytotoxicity was tested by the determination of the cell ATP level by a ViaLightPlus Kit (Lonza, Basel, Switzerland). The method is based on the bioluminescent measurement of ATP, which is present in all metabolically active cells [46]. The bioluminescent method utilizes luciferase, an enzyme that catalyzes the formation of light from ATP and luciferin. The emitted light intensity is linearly related to the ATP concentration, and it is measured using Infinite M200 pro luminometer (Tecan, Männedorf, Switzerland). VFS was tested on human dermis fibroblasts (HDF) and human skin keratinocyte (NCTC2544) cells, which were grown in RPMI 1640 medium plus 10% FCS, penicillin 100 IU/mL and streptomycin 100 μg/mL (cRPMI) overnight to reach confluence (95%). We decided to assess the cytotoxic effect on these two human cell lines, as they represent the main cell types constituting the skin, the target site for the intended future application of VFS. Monolayer cells were treated for 0.5, 1, 4 and 24 h at 37 °C with increasing concentrations of VFS (250, 1–5–25–50–75–90%) prepared in cRPMI. After incubation, plates were left to cool at room temperature for 10 min, and then the Cell Lysis Reagent was added to each well to extract ATP from cells. Next, the AMR Plus (ATP Monitoring Reagent Plus) was added, and after 2 min, the luminescence was read using the microplate luminometer Infinite M200 pro (Tecan).

### 2.9. Effect of VFS on the Regenerative Capacity of Human Dermal Fibroblasts

Regenerative activity has been evaluated as previously described with some modifications [48]. Human dermal fibroblasts (HDF, 106-05A Sigma Aldrich-Merck Life Science) were seeded in a Micro-Insert 4-Well µ-Dish 35 mm (80406 Ibidi, GmbH, Martinsried, Germany) at a concentration of 2 × 10^5^/mL (final volume 15 µL). At confluence, around 95%, the insert was removed, and cells were treated with different concentrations of VFS (1–25–75% *v*/*v*). The growth was monitored by measuring the distance between the two sides of the monolayers at 0 h, 24 h, 48 h and 72 h under an inverted microscope (Nikon Eclipse TF2000-S microscope; Nikon, Milan, Italy) at 20× magnification. The regenerative activity was calculated using the following formula: measured distance of cell migration from starting point in mm at 24, 48, or 72 h × 100/measured distance of cell migration from the starting point in mm at time 0. Wound healing assays with VFS were conducted on HDFs, as they represent the primary cell type involved in extracellular matrix repair and remodeling processes.

### 2.10. Statistical Analysis

Results are expressed as mean ± standard deviation (SD). Significance was tested by means of Student’s two-tailed *t*-test. *p* < 0.05 was considered significant.

## 3. Results

### 3.1. V. filiformis Supernatant VFS Effect on Growth Kinetics of Bacteria

**Figure 1:**
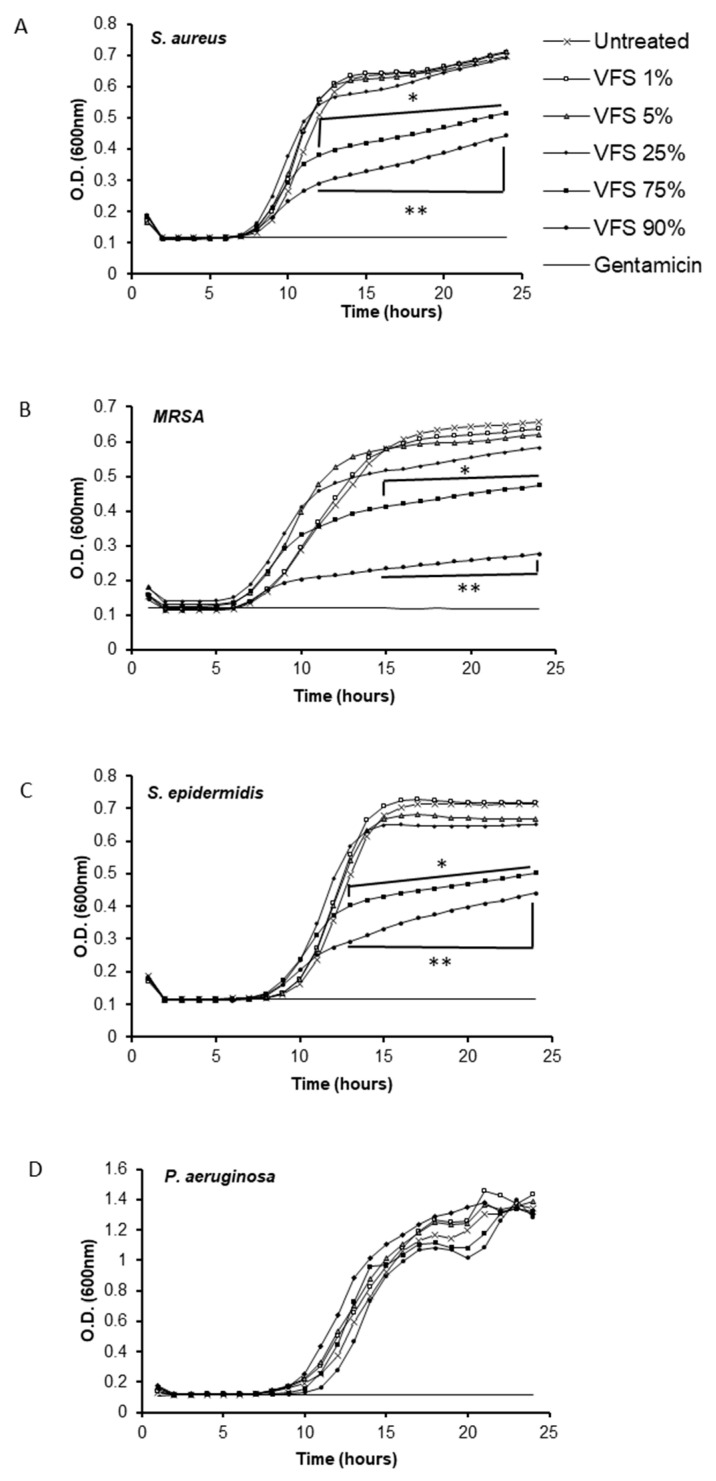
Kinetics of *S. aureus* (**A**), MRSA (**B**), *S. epidermidis* (**C**), and *P. aeruginosa* (**D**) in the presence or absence of the indicated percentages of VFS. Gentamicin (2.5 µg/mL) was used as positive control. Data represent the mean of two independent experiments performed in triplicate. * *p* < 0.01, ** *p* < 0.001 (VFS-treated versus untreated).

The effect of VFS on the growth of Gram-positive bacteria *S. aureus*, the clinical isolate strain MRSA, *S. epidermidis* and the Gram-negative bacteria *P. aeruginosa* was evaluated by spectrophotometry in the presence of different percentages of VFS (Figure 1).

As shown in Figure 1, VFS at 1%, 5%, and 25% does not substantially alter the growth kinetics of *S. aureus* compared with the untreated control. In contrast, higher concentrations (75% and 90%) significantly reduce bacterial proliferation over the 24 h incubation period, with effects comparable to the gentamicin control. A similar pattern is observed for MRSA. Low VFS concentrations (1–25%) do not affect growth, whereas 75% and especially 90% markedly inhibit bacterial expansion. The inhibitory effect at 90% approaches that of gentamicin, indicating strong susceptibility of MRSA to high VFS doses. *S. epidermidis* shows the same dose-dependent response: minimal or no inhibition at 1–25%, followed by a significant reduction in growth at 75% and 90%. The highest concentration produces an inhibition profile similar to the antibiotic control. In contrast to the Gram-positive species, *P. aeruginosa* exhibits no significant growth reduction at any VFS concentration tested. Only gentamicin effectively suppresses its proliferation, confirming the intrinsic resistance of this Gram-negative bacterium to VFS.

### 3.2. Effect of VFS on Biofilm Formation and Dispersal

**Figure 2:**
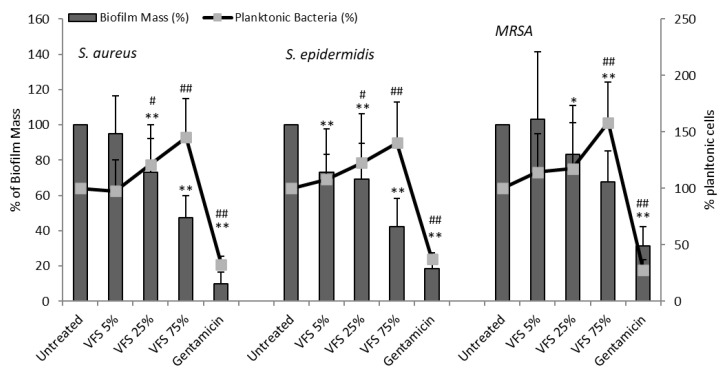
Inhibition of biofilm formation by VFS on *S. aureus*, *S. epidermidis* and MRSA. Data are presented as % of increase or decrease compared to untreated bacteria (100). Data represent the Mean ± SD of at least three independent experiments performed in triplicate. * *p* < 0.05, ** *p* < 0.001 (biofilm mass, VFS-treated bacteria versus untreated); # *p* < 0.05, ## *p* < 0.001 (planktonic bacteria, VFS-treated bacteria versus untreated).

To analyze the anti-biofilm properties of VFS, we tested both the capacity of the supernatants to interfere with staphylococci biofilm formation on abiotic surfaces and their activity on preformed biofilms. Given the absence of antimicrobial activity of VFS on *P. aeruginosa*, the anti-biofilm activity has not been tested. For biofilm formation, bacteria were seeded in 96-well plates in the presence of different concentrations of VFS (5%, 25%, 75%) and cultured under static conditions for 24 h at 37 °C; for biofilm dispersal, bacteria were first cultured for 24 h to allow the formation of biofilms and then treated with VFS. The biofilm mass was assessed by crystal violet staining. VFS was able to inhibit biofilm formation of all tested bacteria at the concentrations of 25% and 75%; moreover, the concentration of 5% was effective in inhibiting *S. epidermidis* biofilm formation (Figure 2).

While the results observed for VFS 75% are not surprising because VFS 75% is able to decrease the growth of *Staphylococcus* cells, VFS 25% showed a good anti-biofilm activity. Moreover, VFS 5% was able to reduce the biofilm mass of *S. epidermidis*. To confirm that the observed results were due to the anti-biofilm properties of the VFS and not to the inhibition of bacterial growth, the planktonic bacterial biomass was evaluated by optical density (O.D. 600 nm). Interestingly, the decrease in biofilm mass was accompanied by a significant increase in the planktonic form compared to the untreated cells. Of interest, the anti-biofilm effect of VFS was also detected on drug-resistant MRSA. Gentamicin inhibited biofilm formation and bacterial growth. As expected, the results underline the effect of VFS on the mechanism of biofilm development; in particular, the increase in the planktonic biomass suggests that VFS could have anti-adhesive activity and does not interfere with bacterial growth.

**Figure 3:**
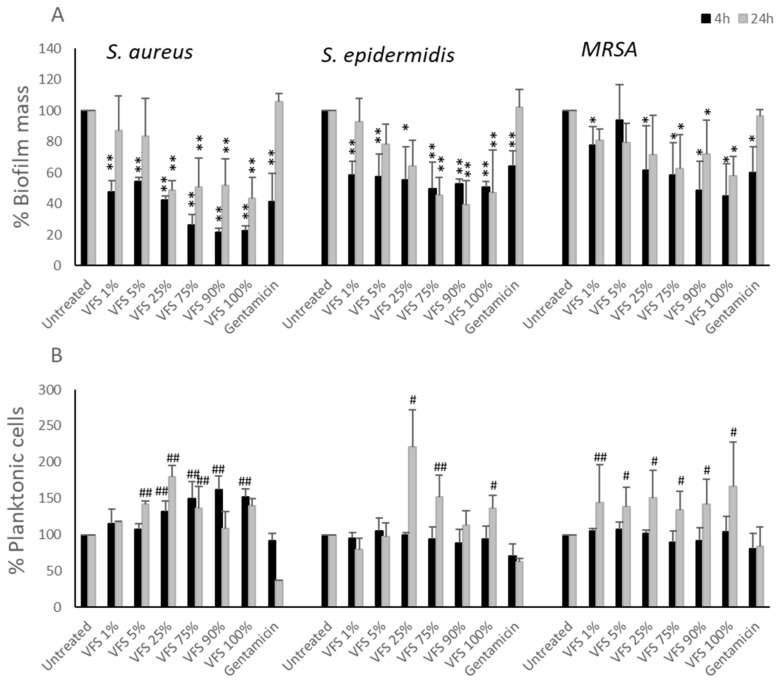
Dispersal activity of VFS on pre-formed biofilms (**A**) and planktonic bacteria (**B**) of *S. aureus*, *S. epidermidis* and MRSA at 4 and 24 h. Data are presented as % of increase or decrease compared to untreated bacteria (100). Data represent the Mean ± SD of three independent experiments performed in triplicate. * *p* < 0.05, ** *p* < 0.001 (biofilm mass, VFS-treated bacteria versus untreated); # *p* < 0.05, ## *p* < 0.001 (planktonic bacteria, VFS-treated bacteria versus untreated).

VFS was able to significantly reduce the mass of preformed biofilms of *S. aureus*, *S. epidermidis* and *MRSA* both after 4 h and after 24 h of treatment (Figure 3). At low VFS concentrations (1–5%), the biofilm mass remains comparable to the untreated control at both 4 h and 24 h, indicating minimal interference with early adhesion or biofilm maturation. Instead, at intermediate concentrations (7.5–10%), a clear reduction in biofilm biomass is observed, more pronounced at 24 h, suggesting that VFS interferes with biofilm consolidation and matrix stability. In parallel with the reduction in biofilm mass, VFS induces a dose-dependent increase in planktonic cells, particularly evident at 7.5% and 10%. This inverse trend indicates that VFS does not simply kill biofilm-embedded bacteria but rather promotes detachment or prevents stable adhesion, shifting the bacterial population toward the planktonic state.

Gentamicin produces the strongest reduction in biofilm mass and drastically reduces planktonic cells due to its bactericidal activity, thereby serving as the positive control for biofilm inhibition.

### 3.3. Antioxidant Activity

**Figure 4:**
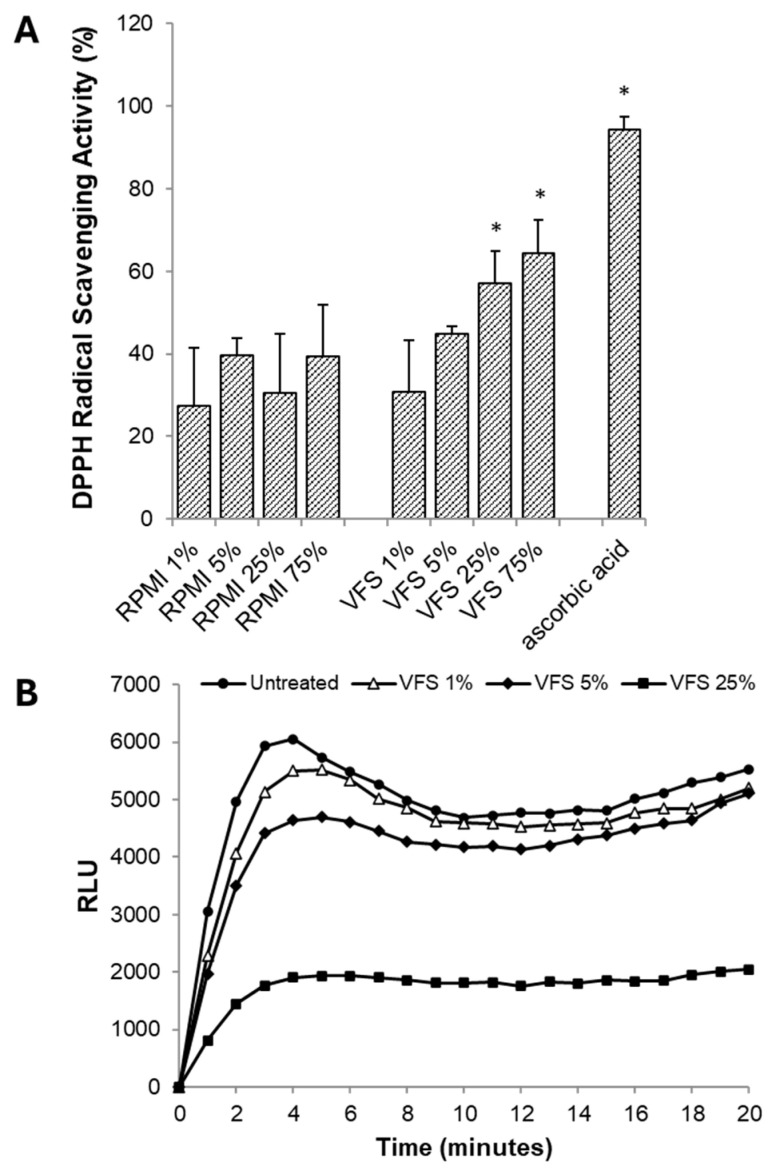
Antioxidant activity of VFS. (**A**) The antioxidant activity of 1%, 5%, 25% and 75% VFS was tested by DPPH assay. Results are expressed % of DPPH scavenging activity. Ascorbic acid (100 µg/mL) was used as positive control. Data represent the Mean ± SD of two independent experiments performed in triplicate. (**B**) The effect of the indicated concentrations of VFS on ROS production by human PMN was evaluated by luminol chemiluminescence assay after PMA stimulation. Data are expressed as RLU and are representative of two independent experiments with similar results performed in triplicate. * *p* < 0.005 (1%, 5%, 25%. 75% VFS versus 1%, 5%, 25%, 75% RPMI).

The antioxidant capacity of *V. filiformis* supernatant was tested both in terms of neutralization of the free DPPH radical and reduction of total ROS produced from PMA-stimulated human PMN. Results shown in Figure 4A highlight that VFS at a concentration of 25% and 75% holds scavenging activity against DPPH.

Upon stimulation with PMA, untreated PMNs exhibit a robust oxidative burst, as expected for this positive activation control. The presence of 1% and 5% VFS concentrations does not significantly alter ROS generation, indicating that these doses do not interfere with PMA-induced activation of the oxidative machinery. In contrast, VFS at 25% markedly reduces ROS production, resulting in a substantial attenuation of the chemiluminescent signal. This reduction demonstrates that higher VFS concentrations can effectively dampen the oxidative response of PMN, suggesting an antioxidant ROS-scavenging effect at the cellular level.

### 3.4. Regenerative Activity

**Figure 5:**
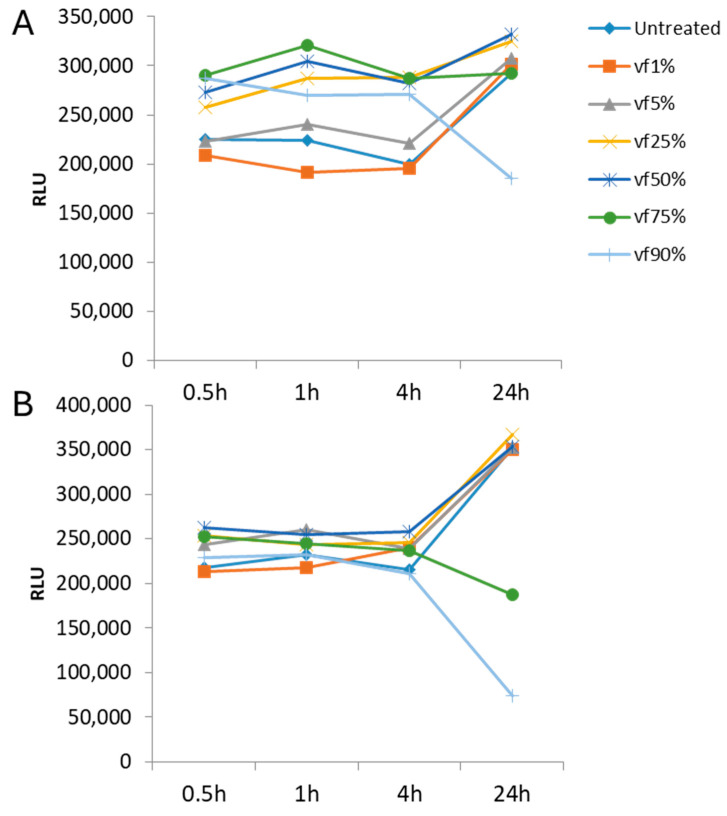
Cytotoxicity of VFS on human dermis fibroblasts (HDF) (**A**) and human keratinocytes NCTC 2544 (**B**). Results are expressed as RLU of live cells. The results are expressed as mean of two independent experiments conducted in triplicate.

**Figure 6:**
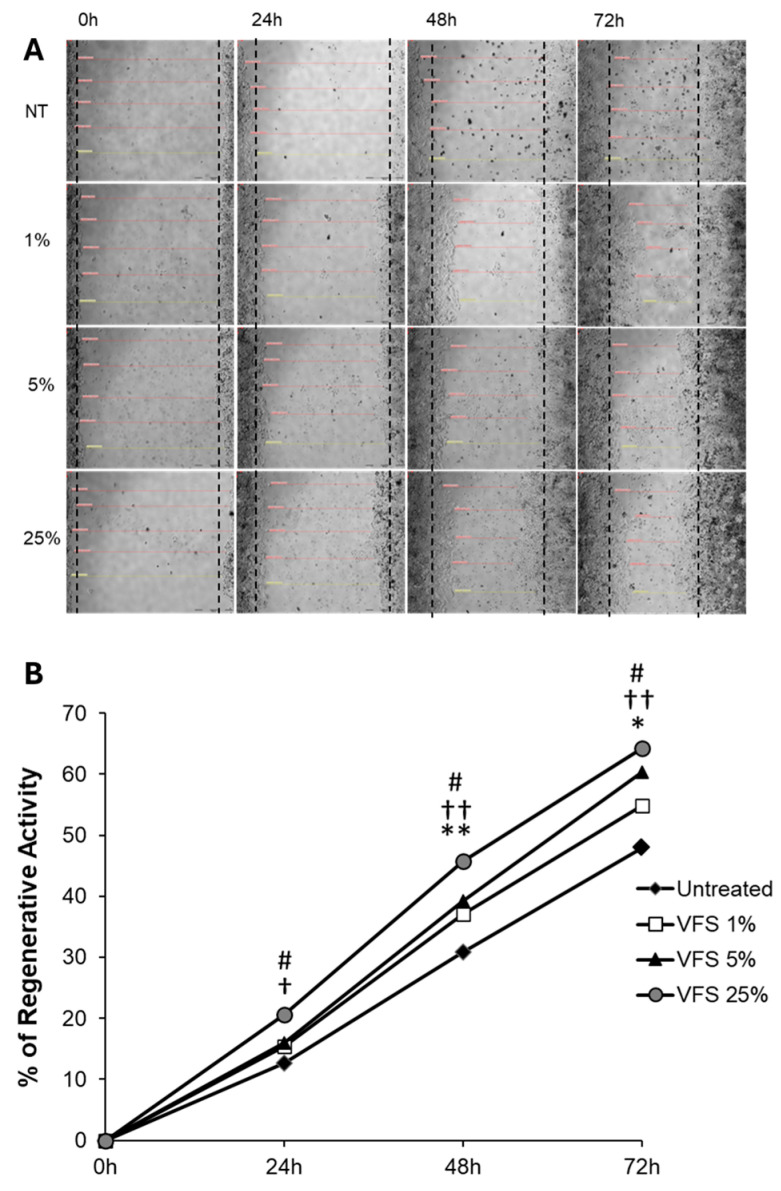
Regenerative activity of VFS. (**A**) Photo-micrographs of HDF cells at different time points cultured in the presence/absence of different percentages of VFS. The red lines and the yellow line in each microscopy image correspond to the measurements performed for each sample. Black vertical dashed lines were inserted at the point of minimal distance between the edges of the treated cell monolayer. (**B**) % of regenerative activity of VFS on HDF cells. Data are expressed as % of regenerative activity of VFS-treated cells compared to untreated cells and are presented as Mean ± SD of 20 measurements taken for each treatment time point in two independent experiments. * *p* < 0.05, ** *p* < 0.01 (1%VFS-treated cells versus untreated cells), † *p* < 0.01, †† *p* < 0.001 (5%VFS-treated cells versus untreated cells), # *p* < 0.001, (25%VFS-treated cells versus untreated cells).

In the first series of experiments, the cytotoxic effect of VFS on cell lines of HDF and human keratinocytes NCTC2544 was evaluated to determine the concentration of VFS for regenerative activity. Figure 5 shows the relative light unit (RLU) values for HDF (Figure 6A) and NCTC2544 cells (Figure 6B) treated with different concentrations of VFS. In both cell lines, a cytotoxic effect was detected only at the highest concentration (90% VFS), starting from 4 h of exposure. Taken together with the regenerative activity data, these findings indicate that treatment with 25% VFS achieves the greatest regenerative response without inducing any cytotoxicity.

To assess the regenerative properties of VFS, HDF cells were cultured in Micro-Insert 4-Well µ-Dish 35 mm until confluence was reached. After removal of the insert, cell migration in the presence of different VFS concentrations was monitored by measuring the distance between the two edges of the monolayer for up to 72 h. As shown in Figure 6, VFS stimulated HDF growth in a dose-dependent manner, with the highest increase observed at 25% VFS (+16.24% compared to untreated cells).

Our results demonstrate that VFS promotes cell proliferation and accelerates wound closure in an in vitro dermal fibroblast regeneration model, suggesting a potential benefit for the treatment of chronic wounds.

## 4. Discussion

*V. filiformis* cell-free supernatant is able to inhibit cell growth of *S. aureus*, *MRSA* and *S. epidermidis* at high concentrations and to dampen bacterial biofilm formation; this result is in line with the research of Gueniche showing a reduction in *S. aureus* colonization of the skin upon *V. filiformis* lysate treatment [41]. Low concentrations were also effective in inducing the dispersal of mature biofilms even after 4 h of treatment in all tested bacteria, even in MRSA strains often isolated in clinical wounds [49]. The significant increase in bacterial concentrations during both biofilm formation and dispersal observed upon VFS treatment is particularly interesting, highlighting that the anti-biofilm activity of VFS is not due to the inhibition of bacterial growth but to its activity on the extracellular matrix characterizing mature biofilms. Indeed, enhancing the transition of biofilms to the planktonic state could revert the resistance of bacteria embedded in biofilm to the most used antimicrobials.

Excessive amounts of ROS have been shown to characterize chronic wounds and to play a central role in delaying wound healing. VFS was able to reduce total ROS production in a cell human neutrophil-based assay in a dose-dependent manner. The direct antioxidant capacity of VFS was also confirmed by cell-free DPPH assay. Immune cells characterize the wound microenvironment and play an important role in the initiation of healing. However, chronic wounds are constantly infiltrated by neutrophils, which release excessive amounts of ROS exerting deleterious effects on wound healing, by degrading extracellular matrix proteins and impairing the function of dermal fibroblasts and keratinocytes [50]. The potency of VFS is attributable to the combined action of all their components. However, several studies have highlighted how *V. filiformis* produces a particular variant of hemoglobin, which possesses interesting properties. It exhibits an exceptionally high oxygen dissociation rate several hundred times higher than that of eukaryotic hemoglobins (VHb). This kinetic property allows VHb to function as an oxygen facilitator rather than an oxygen carrier, rapidly releasing oxygen and efficiently delivering it to terminal respiratory oxidases. VHb enhances cellular respiration, ATP production, and NAD^+^ regeneration through promoting oxidative phosphorylation under oxygen-limited conditions. These properties make VHb widely applicable in improving biosynthetic processes with enhanced growth and increased product yields [51].

This property enhances cellular respiration, ATP production, and NAD^+^ regeneration, thereby improving biosynthetic processes under oxygen-limited conditions and increasing growth and product yield. As further evidence, Mahé et al. (2006) demonstrated the induction of intracellular MnSOD (manganese superoxide dismutase), a major inducible free-radical scavenger of the skin, in normal human dermal fibroblasts and epidermal keratinocytes following *V. filiformis* treatment [38]. Our results on ex vivo human PMN further confirm the antioxidant properties of *V. filiformis*, shedding light on the mechanisms underlying its beneficial effects on skin diseases.

Clinical studies from Gueniche and Seite have clearly highlighted the beneficial effects (lysate including membrane and cytosol) of *V. filiformis* on skin diseases. Moreover, topically applied *V. filiformis* extract has demonstrated probiotic activity by restoring skin barrier function, stimulating the immune response, and reducing infection rates [47,48]. However, while their work involved the use of bacterial lysates and biomass respectively, we only focused on cell-free bacterial-conditioned medium.

The use of VFS has also been shown to promote the proliferation of skin cell lines such as keratinocytes and human fibroblasts. Our results on the regenerative potential of VFS were obtained using an in vitro assay, an approach that has also been employed in other studies to preliminarily demonstrate the ability of different postbiotics to enhance skin regeneration. A recent study evaluated the wound-healing potential of postbiotics derived from *Limosilactobacillus reuteri* EIR/Spx-2. Treatment with postbiotics led to a significant increase in L929 fibroblast cell proliferation (23.82% ± 2.11%); moreover, when applied to scratched fibroblast monolayers, postbiotics markedly accelerated re-epithelialization (66.78% ± 3.74%) [52]. The postbiotic obtained from *Latilactobacillus curvatus* BGMK2-41 promotes wound healing by stimulating keratinocyte migration, as confirmed by scratch assay, and by enhancing the expression of tight junction proteins [53].

Our findings on the ability of VFS to enhance the regeneration of HDF monolayers, together with the absence of cytotoxicity, support the use of the *V. filiformis* supernatant as a promising treatment to accelerate wound healing.

Current approaches for the treatment of chronic wounds include negative pressure wound therapy and hyperbaric oxygen therapy, aimed at clearing the wound of exudate, increasing tissue perfusion and promoting granulation tissue formation, as well as PDGF, Tβ4 and angiotensin, which are used to promote angiogenesis and stimulate cell proliferation [54].

All this evidence makes VFS a potentially effective alternative for the prevention and treatment of chronic wounds, a skin condition in which both microbial biofilm control and stimulation of tissue regeneration are essential.

It has been observed that preparations not containing live microorganisms, but only non-replicating bacterial products, exhibit active properties comparable to those of probiotics [55]. *V. filiformis* extract VFE was employed as an industrial raw material in the development of numerous commercial cosmetic formulations; when applied to the skin, VFE-based products exhibit properties such as reducing inflammation, enhancing skin defenses, and strengthening the skin barrier [39]. An emollient enriched with *V. filiformis* biomass and thermal spring water has shown greater clinical efficacy in the treatment of patients with AD lesions compared to another reference product [35]. The combination of VVMW and extract of *V. filiformis* provides several benefits when applied to the skin: it enhances skin barrier function by promoting epidermal differentiation and the formation of tight junctions, boosts biochemical protection through up-regulation of antimicrobial peptides, and strengthens cellular immune responses [40]. The efficacy of *V. filiformis* lysate was also observed in vivo in NC/Nga mice, a model for human AD. Topical treatment of the mice with a lysate solution, during the elicitation phase of skin inflammation, markedly reduced allergen-specific dermatitis, suggesting that it exerts direct immunosuppressive effects [38]. These findings have led to the market introduction of several cosmetic products based on VFE, including Aqua Posae Filiformis (La Roche-Posay), which is used to rebalance the skin microbiome and strengthen skin barrier functions.

## 5. Future Directions and Limitations of the Study

The study provides important findings, but it has limitations due to the lack of investigation into specific aspects. In vitro systems are limited in their ability to capture polymicrobial interactions and the complexity of in vivo models, particularly the host immune processes involved in wound healing. The translational potential of VFS for chronic wound management is constrained by its ineffectiveness to inhibit *Pseudomonas aeruginosa*, despite showing activity against *Staphylococcus aureus*.

*Staphylococcus aureus* is frequently the dominant pathogen in chronic wounds, including pressure ulcers, venous leg ulcers, and diabetic foot ulcers [56], as well as in postoperative and traumatic lesions. Its ability to colonize intact skin and mucosal surfaces, together with its capacity for biofilm formation [57] and toxin production [58], underlies its persistence as a major cause of both community-acquired and healthcare-associated infections [59]. By contrast, *Pseudomonas aeruginosa* often predominates in wounds with high moisture and impaired barrier function, such as burn injuries, heavily exudative pressure ulcers, and wounds in immunocompromised patients [60]. Its adaptation to humid, low-oxygen environments, combined with intrinsic multidrug resistance [61], makes it a particularly difficult pathogen to manage. Defining the microbial profiles associated with different wound types is therefore critical for guiding antimicrobial strategies and optimizing wound care.

The primary objective of this study was to assess the antibiofilm activity of VFS against Gram-negative bacteria in vitro, with particular emphasis on its relevance in wound environments. Additionally, the study aimed to identify the optimal dilutions of VFS that exhibit both non-cytotoxic and antioxidant properties in the skin cell environment. Based on the observed effects, further investigation in murine models is warranted to evaluate the immune response and therapeutic potential in vivo. Subsequent studies should examine the antibiofilm activity of VFS against other clinically relevant Gram-negative pathogens commonly implicated in chronic wound infections, including *Escherichia coli*, *Klebsiella pneumoniae*, and *Enterobacter* spp., and to assess the in vivo efficacy of VFS using murine models of chronic wounds, particularly those co-infected with the aforementioned pathogens. If the results prove promising, the next step would be to develop the postbiotic in a pharmaceutical formulation suitable for topical administration and to evaluate it in a clinical study following the enrollment of subjects with chronic wounds of diverse types and characteristics. The study would be conducted in accordance with the guidelines for the commercialization of biotechnological products established by the regulatory authorities AIFA (Italian Medicines Agency) and EMA (European Medicines Agency).

Although *Vitrescilla*-derived hemoglobin has been widely used as a metabolic engineering tool in microorganisms, plants and animals with no major safety concerns reported to date, a systematic toxicological characterization of purified *Vitrescilla*-derived products in mammals remains limited. Potential risks that warrant formal evaluation include immunogenicity (e.g., anti-VHb antibody formation and cross-reactivity with host hemoproteins), heme-mediated oxidative stress, and off-target effects arising from ectopic expression or unintended tissue distribution in vivo. From a regulatory perspective, any *Vitrescilla*-derived biologic intended for therapeutic use would be regulated as a novel biologic (e.g., under the FDA’s Center for Biologics Evaluation and Research or the EMA’s advanced therapy/biologic frameworks), requiring a full preclinical package (dose toxicity, safety pharmacology, immunogenicity, and, where relevant, genotoxicity and reproductive toxicity), robust Chemistry, Manufacturing, and Controls characterization, and phase-appropriate clinical safety monitoring. Taken together, these considerations highlight that, despite promising functional data, the safety profile of *Vitrescilla*-derived compounds cannot be assumed and must be established through targeted toxicology and regulatory grade studies—an important limitation of the current work that should be addressed in future development [62].

## 6. Conclusions

These preliminary results are highly encouraging and underscore the significant potential of VFS, which, in this study, was employed for the first time as an antibiofilm agent. Our findings suggest that VFS can target microbial communities involved in chronic and acute skin conditions, paving the way for its potential use beyond conventional antimicrobial approaches. We anticipate that these results, combined with further preclinical and clinical investigations, will support the development of VFS-based postbiotic formulations suitable for both clinical and cosmetic applications. Such formulations could offer a novel strategy for the management of specific skin disorders, particularly those characterized by biofilm-associated infections or microbial dysbiosis, thereby bridging the gap between experimental research and practical therapeutic interventions.

## Data Availability

The original contributions presented in this study are included in the article. Further inquiries can be directed to the corresponding author.

## Abbreviations

The following abbreviations are used in this manuscript:

VFS	*Vitreoscilla filiformis* supernatant
VFE	*Vitreoscilla filiformis* extract
MRSA	Methicillin-resistant *Staphylococcus aureus*
CoNS	Coagulase negative staphylococci
ROS	Reactive oxygen species
DPPH	2,2-diphenyl-1-picrylhydrazyl
AD	Atopic Dermatitis
EPS	Extracellular Polymeric Substances
RPMI	Roswell Park Memorial Institute Medium
ATCC	American Type Culture Collection
PMN	Polymorphonuclear Cells
MnSOD	Manganese superoxide dismutase
VVMW	Vichy volcanic mineralizing water
HDF	Human dermal fibroblast
